# Editorial: Women in science: Interventions for rehabilitation

**DOI:** 10.3389/fresc.2022.1008741

**Published:** 2022-08-25

**Authors:** Maryam Zoghi, Maria Rubega, Joyce Fung

**Affiliations:** ^1^ Discipline of Physiotherapy, Institute of Health and Wellbeing, Federation University, VIC, Australia; ^2^ Section of Rehabilitation, Department of Neuroscience, University of Padova, Padova, Italy; ^3^ School of Physical and Occupational Therapy, Faculty of Medicine and Health Sciences, McGill University, Montreal, QC, Canada

**Keywords:** postural balance, wearable sensor, sialorrhea, cerebral palsy, spinal cord injury

**Editorial on the Research Topic** Women in science: Interventions for rehabilitation BY Zoghi M, Rubega M, Fung J. (2022) Front. Surg. 3: 1008741. doi: 10.3389/fresc.2022.1008741

We are thrilled to present the Frontiers in Rehabilitation Sciences “Women in Science: Interventions for Rehabilitation” series of article collection.

There are fewer than 30% of women researchers in the world. Women and girls are discouraged from studying Science, Technology, Engineering, and Math (STEM) fields due to long-standing prejudices and stereotypes. Despite men and women publish at similar rates and have similar career outcomes based on total number of publications, Huang et al. (1), the gender gap further widens in academic positions, Casad et al. (2). Science and gender equality are essential to ensure sustainable development as highlighted by UNESCO. Gender equality must be promoted, stereotypes must be challenged, and girls and women should be encouraged to pursue STEM careers in order to change traditional mindsets.

A key feature of this Research Topic is the diversity of research, mainly by women, undertaken across the entire spectrum of interventions for rehabilitation research, and how advances in theory, experimentation, and methodology are applied to compelling issues. The works presented here span from wearable sensors application for tracking and classifying human movements, Lang et al. (2022), to interventions for improving postural balance and muscle control in people with spinal cord injury, Nascimento and Boffino (2022), Sato-Klemm et al. (2022), and for sialorrhea in cerebral palsy, Marquez Vazquez et al. (2022).

Upper limb functions can be significantly affected after a stroke. About eighty-five percent of stroke survivors suffer from upper limb dysfunctions, Kwakkel et al. (3), which can affect their daily activities considerably, Aparicio et al. (4). Assessing the impact of these dysfunctions on daily activities can be challenging. Lang et al. (2022) used bilateral, wrist-worn accelerometers to identify categories (low, medium and high) of upper limb performance during a 24-h period in 135 stroke patients and 76 neurologically intact adults. They identified a 5-cluster categorization that included 5 upper limb performance variables. Two groups in this cluster were identified in the neurologically intact adults as having a normal level of activity (moderate to high activity level) with full integration of the upper limb performance into daily activities in life. The middle group in the stroke cohort had a moderate level of activity with moderate integration of the upper limb performance in daily activities and the last two groups in the stroke cohort had a low level of activity with minimal to rare integration of the upper limb performance in daily activities.

Traumatic spinal cord injuries have elicited a global quest in the promotion of neuroplasticity and restoration of movements and functions with novel neuromodulation and neurorehabilitation strategies. Galvanic vestibular stimulation (GVS) has recently gained attention in its potential to activate vestibulospinal and reticulospinal pathways as well as the propriospinal neuronal network Sayenko et al. (5). In a non-linear system, such as the vestibular system, low residual functions can be amplified through stochastic resonance when imperceptible noise is added to the stimulation. The case study reported by Nascimento and Boffino (2022) demonstrated that 10 sessions of GVS applied with random noise stimulation, in addition to neurorehabilitation, promoted significant functional recovery in a tetraplegic individual with a chronic spinal cord injury who was discharged from rehabilitation 2 years prior to the study. The impairment classification according to the American Spinal Injury Association changed from level A (complete) to level C (incomplete) with the motor levels improving from C5 bilaterally to C6 (R) and C7 (L). With the added strength in wrist extensors (C6) and elbow extensors (C7), as well as enhanced trunk and postural control, bed mobility and transfers also improved. This proof-of-principle case study reveals that noise-added GVS can be used not only for non-invasive neuromodulation but also as a potential biomarker for adaptive neuroplasticity following spinal lesions.

Spinal cord injury (SCI) has damaging consequences for the physical, social and work well-being. Managing the complications of SCI is key to address all facets of the patient’s injury, Ahuja et al. (6). Sato-Klemm et al. (2022) assessed knowledge, attitudes and practice of pelvic floor muscle training (PFMT) in SCI via an online survey. They investigated in 153 participants (62% female, [46.5±14.3] y): their knowledge in PFMT; their access to PFMT and their ability in contracting pelvic floor muscles. Despite people with SCI were aware of PFMT and had favorable attitudes toward it, few had actually practiced PFMT. The authors suggest future studies to explore the benefit of PFMT for people with this kind of injury.

Cerebral palsy (CP) is a neurological condition with important sequelae in sensory and motor impairments. Sialorrhea and sleep problems are common comorbidities that have recently been revealed to be prevalent in children with CP. Sialorrhea results from failed clearance and removal of saliva from the oral cavity, most often associated with impaired swallowing, and lead to not only medical problems such as pneumonia, but also psychosocial problems for the family and the developing child. In a longitudinal clinical trial involving 134 children with CP, Marquez Vazquez et al. (2022) found a correlation between the severity of sialorrhea and sleep disorders with dysphagia, which can be alleviated with AbobotuliniumtoxinA or oromotor therapy. Systematic screening of sleep disorders and swallowing efficacy in CP children presented with siaolorrhea, and intervention with oromotor therapy combined with AbobotuliniumtoxinA in severe cases are recommended.

Since the journey for gender equality in academia, particularly in STEM, remains long and arduous, we encourage all readers to play their own part in promoting original works contributed by women researchers.

## References

[1] Huang J Gates AJ Sinatra R Barabási AL. Historical comparison of gender inequality in scientific careers across countries and disciplines. Proc Natl Acad Sci (2020) 117:4609–16. 10.1073/pnas.1914221117 32071248 PMC7060730

[2] Casad BJ Franks JE Garasky CE Kittleman MM Roesler AC Hall DY, et al. Gender inequality in academia: problems and solutions for women faculty in stem. J Neurosci Res (2021) 99:13–23. 10.1002/jnr.v99.1 33103281

[3] Kwakkel G Kollen BJ van der Grond J Prevo AJ. Probability of regaining dexterity in the flaccid upper limb: impact of severity of paresis and time since onset in acute stroke. Stroke (2003) 34:2181–86. 10.1161/01.STR.0000087172.16305.CD 12907818

[4] [Dataset] Aparicio HJ Beiser A Himali J Satizabal C Pase M Romero J, et al. Temporal trends in stroke incidence in the young in the framingham study (i2. 003) (2016).

[5] Sayenko D Atkinson D Mink A Gurley K Edgerton V Harkema S. Vestibulospinal and corticospinal modulation of lumbosacral network excitability in human subjects. Front Physiol (2018) 9:1746. 10.3389/fphys.2018.01746 30574093 PMC6291495

[6] Ahuja CS Wilson JR Nori S Kotter M Druschel C Curt A, et al. Traumatic spinal cord injury. Nat Rev Dis Primers (2017) 3:1–21. 10.1038/nrdp.2017.18 28447605

